# Forensic Microbiome Database: A Tool for Forensic Geolocation Meta-Analysis Using Publicly Available 16S rRNA Microbiome Sequencing

**DOI:** 10.3389/fmicb.2021.644861

**Published:** 2021-03-23

**Authors:** Harinder Singh, Thomas Clarke, Lauren Brinkac, Chris Greco, Karen E. Nelson

**Affiliations:** ^1^J. Craig Venter Institute, Rockville, MD, United States; ^2^Noblis, Reston, VA, United States; ^3^GeneDX, Gaithersburg, MD, United States

**Keywords:** microbiome, forensic, geolocation, 16S rRNA, database

## Abstract

The human microbiome has been proposed as a tool to investigate different forensic questions, including for the identification of multiple personal information. However, the fragmented state of the publicly available data has retarded the development of analysis techniques and, therefore, the implementation of microbiomes as a forensic tool. To address this, we introduce the forensic microbiome database (FMD), which is a collection of 16S rRNA data and associated metadata generated from publicly available data. The raw data was further normalized and processed using a pipeline to create a standardized data set for downstream analysis. We present a website allowing for the exploration of geolocation signals in the FMD. The website allows users to investigate the taxonomic differences between microbiomes harvested from different locations and to predict the geolocation of their data based on the FMD sequences. All the results are presented in dynamic graphics to allow for a rapid and intuitive investigation of the taxonomic distributions underpinning the geolocation signals and prediction between locations. Apart from the forensic aspect, the database also allows exploration and comparison of microbiome samples from different geolocation and between different body sites. The goal of the FMD is to provide the scientific and non-scientific communities with data and tools to explore the possibilities of microbiomes to answer forensic questions and serve as a model for any future such databases.^1^

## Introduction

Advances in the depth of DNA sequencing over the last couple of decades, labeled as next generation sequencing (NGS), has greatly expanded the knowledge of the diversity of bacteria living on or within humans (microbiomes). Examinations of human microbiomes *via* multiple methods, including directed sequencing of 16S ribosomes (rDNA genes), allow for an estimation of the taxonomic diversity and the distribution of the contributory bacterial species. Experiments have demonstrated that human microbiomes are constantly interfacing with external microbiomes, both from other people, animals (Song et al., 2013; Misic et al., 2015), and from environments (Flores et al., 2011; Hewitt et al., 2012; Luongo et al., 2017). Studies have also demonstrated that the species makeup of human microbiomes is partially shaped by personal factors, including age (Odamaki et al., 2016), diet (De Filippo et al., 2010; Yatsunenko et al., 2012; David et al., 2014), habits (Moon et al., 2015; Wu et al., 2016), disease state (Peters et al., 2016), and geolocation (Yatsunenko, et al., 2012; Zhang et al., 2015; Lund et al., 2017; Brinkac et al., 2018), with the location on the body the strongest determinant (Human Microbiome Project, 2012). As such, this ability to capture and leverage these differences in the human microbiome presents exciting new possibilities for forensic science (Clarke et al., 2017; Hampton-Marcell et al., 2017), including the possibility of linking specific human subjects to objects and locations in the crime scene (Lax et al., 2015) and determining the country of origin for different samples.

Personal identification using microbial biosignatures is still an emerging field, and additional work is necessary for it to become highly effective in forensic science as would be required to be judicially acceptable as evidence. Single sample studies, while sufficient to identify differences between individuals along with a forensic question, are often too restricted in size and scope, such as only addressing one location or one metadata variable. Likewise, though the number of available microbiome samples are rapidly increasing, the diversity of sampling techniques and a lack of uniformity in reporting the metadata associated with the data retards the attempts to use this data in a meta-analysis.

**Figure 1:**
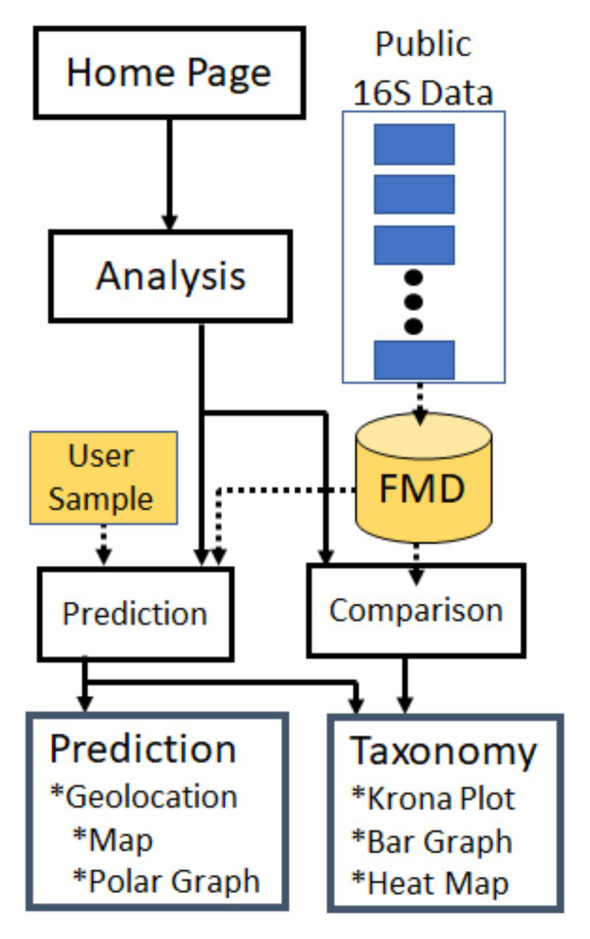
Forensic microbiome database (FMD) and Website Flowchart. Publicly available 16S rRNA sequences were collected from multiple sites, processed, and deposited in the FMD database (detailed in Figure 2). This data can be explored through the FMD website, where it is used to predict the geolocation of user-provided samples (see also Figure 4) and for comparisons of the microbial taxonomic distribution in different geographic and body sites (see also Figure 3).

We have addressed these limitations through the creation of a new database with an associated website that collects and collates publicly available microbiome datasets. The database is populated with ~20,000 human 16S rRNA NGS samples from multiple body sites from various public repositories, which have been subsequently processed using a single pipeline. Apart from sequences, we also capture the metadata associated with the samples including geolocation, healthy or non-healthy status, and other variables. The associated website allows users to compare microbiomes from different geographic locations and body sites, as well as to upload data that can be compared to microbiomes in the database and for which the geolocation of the sample can be predicted (Figure 1). The results from these analyses are provided in dynamic visualizations, which show the taxonomic distribution underpinning the analyses and how the individual samples compare to each other.

## Database Construction

### Data Collection

We surveyed the literature and public repositories for microbiome studies based on 16S rRNA sequencing. Only projects with 16S rRNA sequences sampled from humans with the sampled body site, geographic location, and publishing work all documented were included. Samples of raw sequencing data for each project were downloaded along with available metadata from publicly available databases, including NCBI SRA, EBI, and MG-RAST. The databases include buccal mucosa and stool samples recently collected as part of the forensic microbiome database (FMD) project (PRJNA545251) from adult females (18–26), born and currently living in Barbados (*n* = 32), Santiago (*n* = 32), Pretoria (*n* = 37), and Bangkok (*n* = 60), and described more fully in Clarke et al., *submitted*. Additional metadata values, including age, gender, and healthy/non-healthy status, were used when available either in the public database or in the citing manuscript. The data was processed with the JCVI pipeline based on UPARSE and SILVA database. Diseases such as IBS and Crohn’s disease can have a significant effect on the microbiome (Carroll et al., 2012; Morgan et al., 2012; Zhou et al., 2018). Since disease states can markedly change the microbiome comparison, only samples not explicitly labeled with a disease state are included.

### The 16S rRNA Pipeline

Each project was processed separately, and operational taxonomic units (OTUs) were generated *de novo* from raw 454 or Illumina sequence reads using the UPARSE pipeline (Edgar, 2013). Paired-end reads were trimmed from the adapter sequences, barcodes, and primers prior to assembly. Sequences of low quality and singletons were discarded. Sequences were further subjected to de-replication and chimera filtering during clustering. Mothur (Schloss et al., 2009) was used to report full taxonomies with 100 iterations for the wang classifier (iters = 100) wand, only including sequences where 80 or more of the 100 iterations are reporting similar assignment (cutoff = 80). The RDP classifier in mothur and version 123 of the SILVA 16S ribosomal RNA database (Quast et al., 2013) were used for the taxonomy assignment of OTUs. Rare OTUs or taxa are strongly affected by sequencing errors, and statistical conclusions relying on them are typically unstable (He et al., 2015). The OTUs with less than 10 total reads in each project dataset were considered rare OTUs using the phyloseq (McMurdie and Holmes, 2013) package in R and were removed along with OTUs that were either unknown or unclassed at the genera level. Quality control was also performed on all samples, and the OTUs with samples containing more than 20% of their reads in unknown or unclassified genera or less than 2,000 reads were removed (Amir et al., 2017; Singh et al., 2017). We removed these samples because OTUs with no genera classification will introduce biases in the composition plots, average calculation and impact the prediction module. The trimmed samples were then normalized to their proportion of reads in each OTU and combined into a master OTU table using the phyloseq merge function. All of the microbiome data present in the FMD database are at the genus level. The phyloseq tax_glom function to merge the same genera into one single genera in each separate project was used.

### Database Architecture

Forensic microbiome database is built on Apache HTTP server 2.2 with MySQL server 5.1.47 as the back end and PHP 5.2.9, HTML, and JavaScript as the front end.

### Database Summary

**Figure 2:**
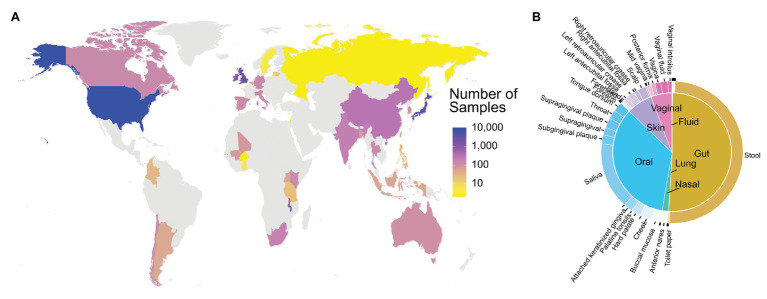
Data Availability in the FMD. The number of samples in the FMD from different countries **(A)** and body sites **(B)**. Only samples from healthy individuals are shown.

The current version of FMD has 20,820 samples from 95 projects with 79 PubMed references. These 96 projects contain 16S rRNA data obtained from 54 different body sites of individuals from 35 different countries, 91 states, provinces or equivalent, and 138 cities. The samples in the database are highly concentrated in developed countries, with the United States (9,492 samples) the most significant contributor in the FMD, followed by Japan (4,054 samples) and the United Kingdom (2,722 samples; Figure 2A). The majority of 16S rRNA data (~50%) was obtained from stool samples, followed by saliva and other oral locations (Figure 2B). Detailed descriptions of the included data are available at http://fmd.jcvi.org/stat.php.

### Web Interface

**Figure 3:**
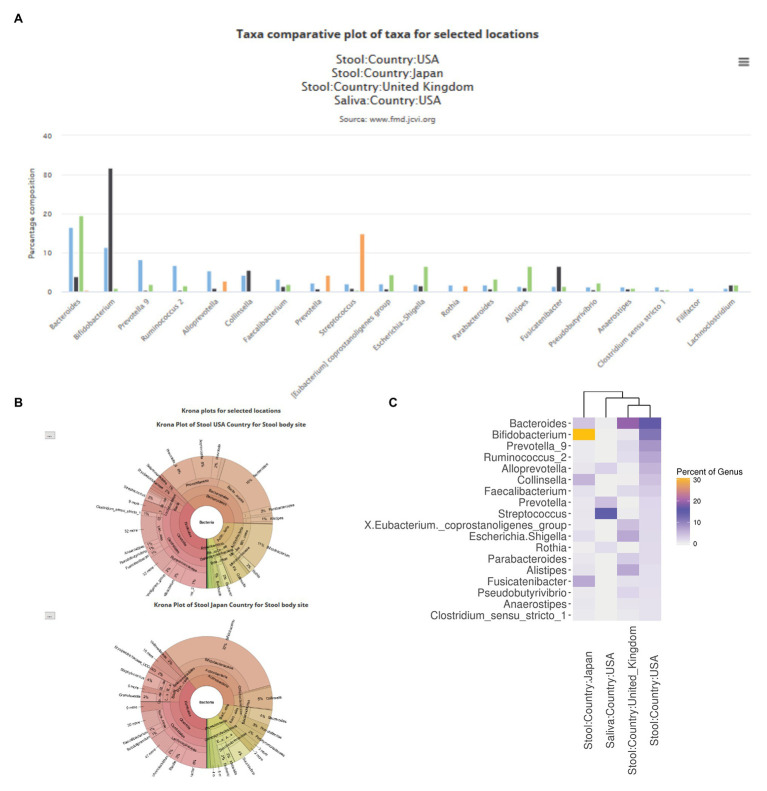
Examples of comparative taxa abundance module in the FMD. The FMD allows for a visual comparison of the taxa abundance of different body sites in different geolocations, which is shown *via* bar charts **(A)**, Krona plots **(B)**, and heatmaps **(C)**. Stool samples from the United States, Japan, and the United Kingdom, as well as saliva samples from the United States, are shown.

The FMD website contains two separates but connected modules. The first allows the user to explore the loaded 16S rRNA data and compare various geolocation and body sites using the processed and loaded data described above. To explore the FMD data, a user can compare the taxonomic abundance profile of individuals’ microbiomes from multiple geolocations and body sites. The first option is a bar plot of the top twenty abundant genera (ranked based on the first selected geolocation in the query) of the selected geolocations (Figure 3A). The second option is the Krona charts of all the selected geolocations (Ondov et al., 2011), with individual Krona charts available for full-screen visualization (Figure 3B). The final option is a heatmap showing the relative abundances of the top ten most abundant genera, ranked similarly to the bar plots, of all the selected geolocations.

**Figure 4:**
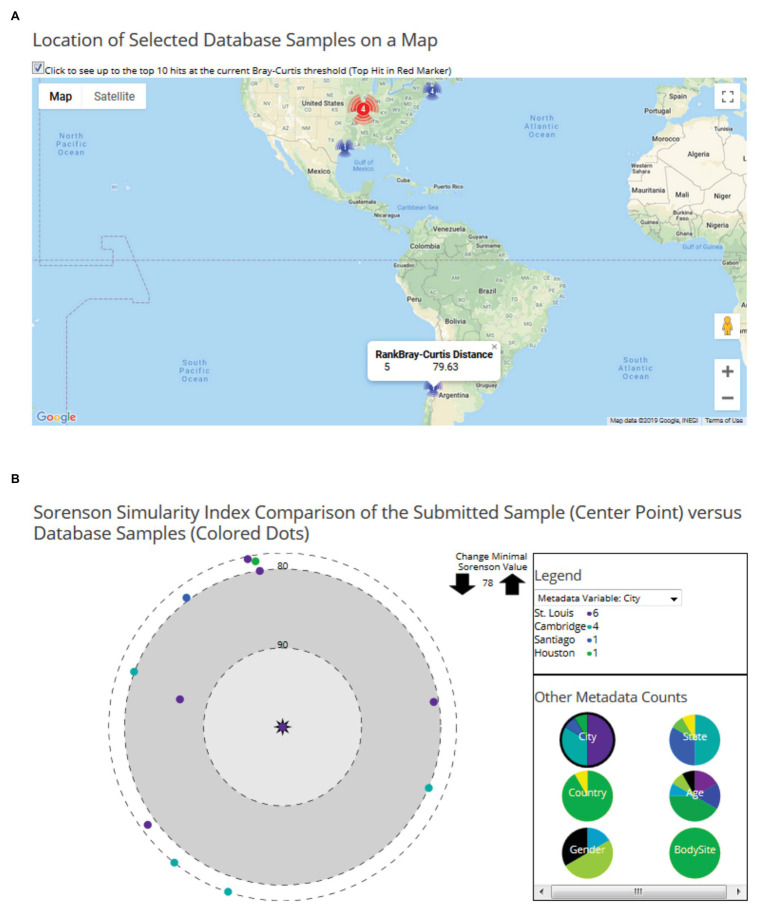
Examples of user data geo-location prediction in the FMD. Geolocation of user-uploaded data is predicted by finding the taxonomically closest FMD samples, which are shown both on a world map, with the number of hits per city shown, **(A)** and as individual samples on a polar graph with their similarity to the sample indicated by the distance to the center **(B)**. 16S rRNA microbiomes obtained from stool samples of an individual residing in St. Louis (Sample ID: SRS015854) is shown here.

In the second module, users can upload their processed microbiome data to predict the potential geolocation of the user-provided data and compare it with any existing geolocation data in FMD. To geolocate the user sample, it is compared against all the samples present in the FMD using the Bray-Curtis distance matrix score, and the results are ranked. The results are visually explorable by both locations and by sample. First, the page displays a world map showing the location and counts of the high-ranked matches, which the site with the top match in a different color (Figure 4A). The distance between the user sample and the high matching samples in the site can be displayed by mousing over the respective site. A second tab contains a sample-level visualization of the Bray-Curtis distances between the user and the FMD samples with distances less than the cutoff points on a polar graph (Figure 4B). The cutoff distance for displayed values can be altered to examine as many sites as desired. The FMD-sample points are colored by metadata values, beginning with geolocation but changeable to age, gender, and body site. The percentage of samples with distances above the cutoff with different values for each metadata variable is also shown. The taxonomic distribution user-submitted samples can also be visualized similarly to the FMD database samples, either with Krona charts or compared with any geographic site data present in the FMD, represented as the average of all the samples of that particular site, as a bar chart and heatmap. A detailed description of the website’s usage can be obtained using the user manual available on the website.^3^

**Figure 5:**
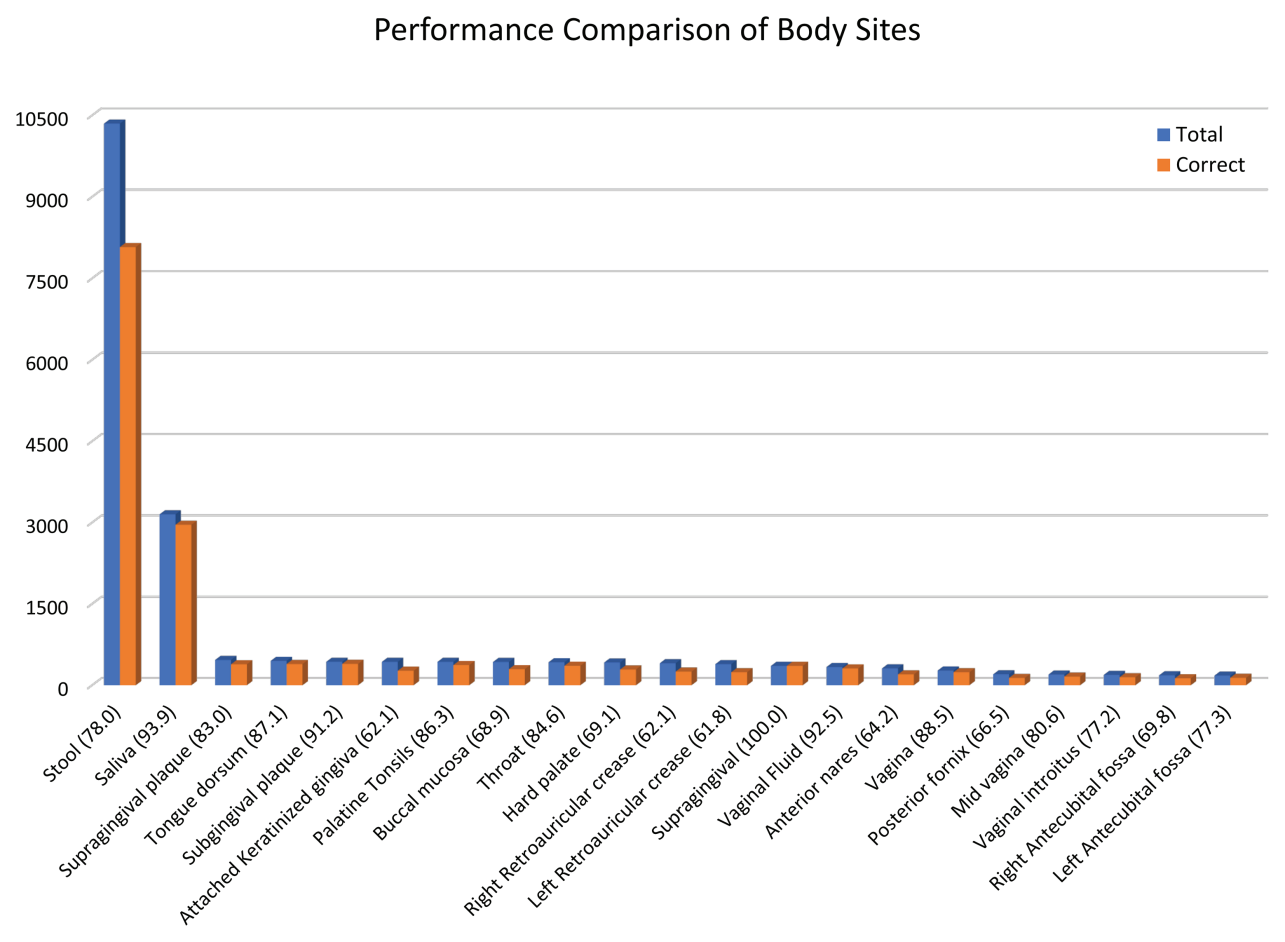
Similarity-based prediction performance of different body sites. A leave-one-out cross-validation of the data was performed using a similarity-based prediction approach. The results for body sites with more than 150 samples are shown. The bar graph shows the total number of samples for a body site and the number of samples with correct geolocation prediction.

To better understand the similarity-based approach’s prediction capability, we performed the leave-one-out cross-validation to estimate the prediction module’s performance. Body sites with more than 150 samples in the database were considered, which constitute 96% of the data. As observed in Figure 5, the overall accuracy is 80.5% for cities, 81.5% for state/region, and 92.1% for countries. The accuracy ranges from 61% for retroauricular crease to 93% for saliva samples (Figure 5). We were able to achieve 78% prediction accuracy for stool samples, which constitute half of the samples collected from all around the world. Further, we explored the impact of similar body sites on the prediction module performance in Supplementary Figure S1. We observed that similar body sites are cross-predicted, i.e., the supragingival samples can be predicted as Subgingival plaque samples and vice versa. There is negligible cross prediction between the oral cavity, skin, vagina, and stool samples which validates the unique microbiome composition of different body sites. Next, we analyzed the remaining incorrect 20.5% samples to understand the impact of distance on incorrect predictions. In the case of incorrectly predicted vagina samples which constitute 13% of all vagina samples, the average distance is ~7,000 km. On average, the incorrect prediction has ~1,000 km distance (Supplementary Figure S2). We examine the incorrect vagina samples that were predicted as stool samples are dominated by the same genus, which suggests either cross-contamination or biological/technical contamination, which explains the considerable variation in incorrect samples’ distance. When we remove the samples where a single genus is more than 60% of microbiome composition, only eight vagina samples were predicted as a stool instead of 35 wrong predictions (Supplementary Figure S3).

## Summary

Numerous studies have identified microbiomes’ potential to be a valuable forensic investigatory tool, but the translation of these findings into legally actionable information remains incomplete. The development of these tools is hindered by multiple limitations of analyzing the data, such as the diversity of formats in which the information is available, the absence of sufficient metadata, and the large amount of data required to generate any tools. The database introduced here begins to address these limitations and can form the backbone for future explorations and generation of a novel technique to tease apart the signals within microbiomes to detect forensic information. The database and the website will facilitate exploration of the taxonomic underpinnings of geolocation signals, both through dynamic explorations of the taxonomic distributions of microbiomes from different geographic locations through comparisons of the data samples in combination with user-supplied metadata. The key limitation of the database is the unavailability of data from many African and Middle east countries apart from few countries from each continent. Since we considered only good quality microbiome data that was not explicitly labeled with a disease state; we were limited to data availability. We hope that in the future, additional data from these regions will be available from the public database and will be added to the FMD database.

The FMD is designed for rapid and intuitable exploration of geolocation signals in the microbiomes using well-documented and computationally inexpensive algorithms. Currently, the database only uses 16S rRNA sequences for the geolocation analysis, and while metagenomic whole genomic sequencing of microbiomes are a rapidly expanding field (Schmedes et al., 2017; Almeida et al., 2019), the analytical tools available to distinguish the geo-position of metagenomes are not as developed. Likewise, machine learning and other reduced taxonomic comparisons are emerging tools for dissecting taxonomic distributions and looking at forensic questions (Johnson et al., 2016; Sarkar et al., 2017), but these have yet to be adapted for a global analysis.

As the state of forensic analysis of microbiomes continues to develop, the FMD is well adapted to address some of the remaining outstanding issues. As previously documented, the body site sampled remains the primary determinant of the taxonomic distribution differences of microbiomes, and multiple body sites have been shown to have a geographic-specific signal (Zhang et al., 2015; Sarkar et al., 2017; Brinkac et al., 2018). By collecting samples from multiple body sites, the FMD currently allows for comparison of the geolocation signals. While current analysis suggests that this signal is not additive across body sites, future analytical techniques might have an amplification effect across body sites that the FMD would capture. Additionally, both the raw information and subsequent analyses can be highly complex and not readily digestible by non-specialists. As the analysis tools increase in complexity, we believe that the summary dynamics figures used by the FMD provide a useful example of how to engage a non-specialist in an analytical examination of the results.

## Data Availability Statement

The datasets presented in this study can be found in online repositories. The names of the repository/repositories and accession number(s) can be found at: http://fmd.jcvi.org/stat.php and http://fmd.jcvi.org/bioproject/PRJNA545251.

## Author Contributions

HS and TC generated the data, designed/developed the database, and wrote the manuscript. LB curated the data, participated in manuscript writing, and designed the database and project. CG generated the data. KN participated in design and implementation of the project. All authors contributed to the article and approved the submitted version.

### Conflict of Interest

CG was employed by company GeneDX.

The remaining authors declare that the research was conducted in the absence of any commercial or financial relationships that could be construed as a potential conflict of interest.

## Footnotes

^3^http://fmd.jcvi.org/help.php
