# Supplementary figures and images for: Forensic Microbiome Database: A Tool for Forensic Geolocation Meta-Analysis Using Publicly Available 16S rRNA Microbiome Sequencing
Source: Front Microbiol. 2021 Mar 23;12:644861. doi: 10.3389/fmicb.2021.644861 (PMC8022992; doi:10.3389/fmicb.2021.644861)

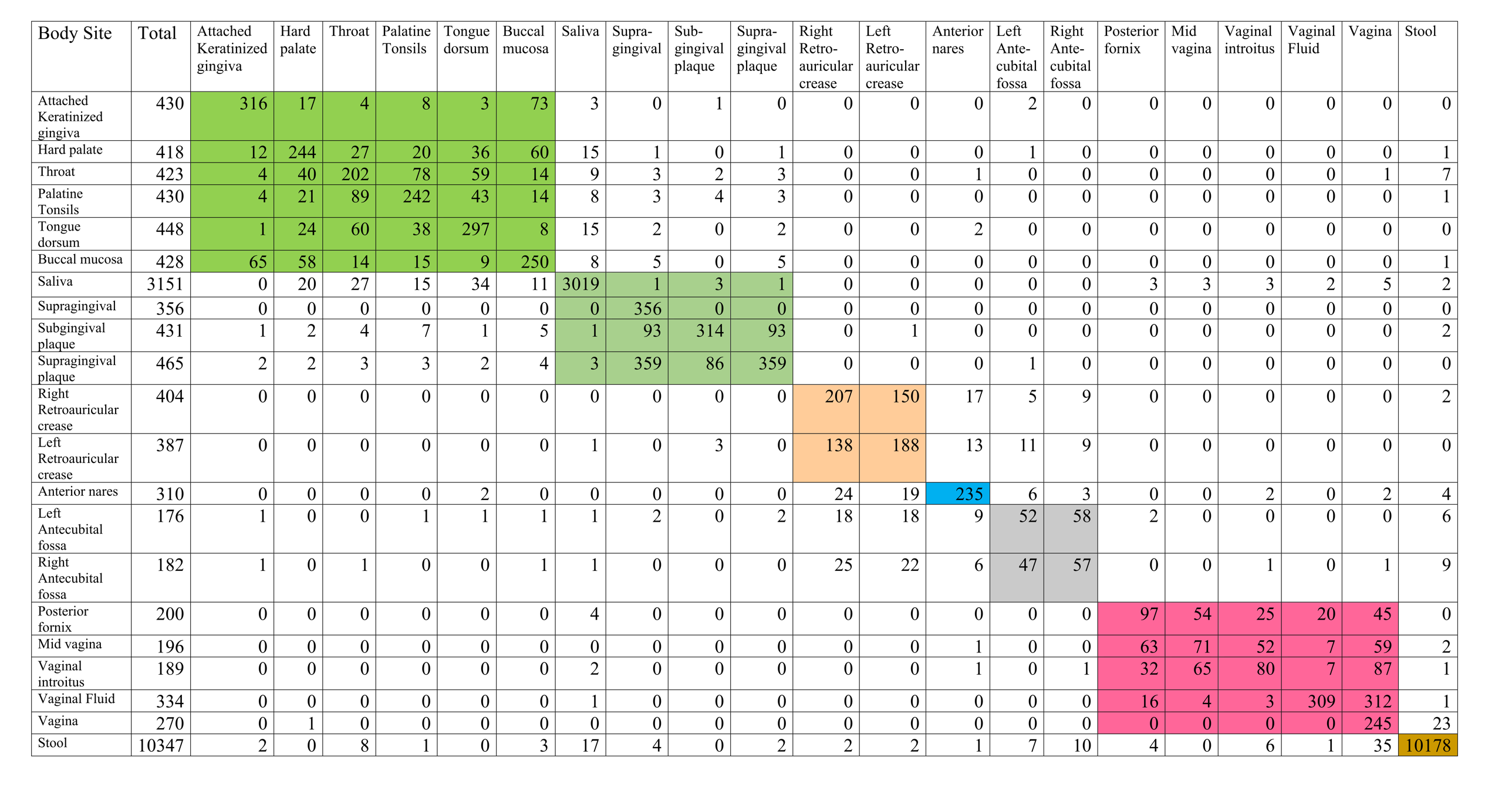

Supplement: Supplementary file 1 [file Image_1.tiff]

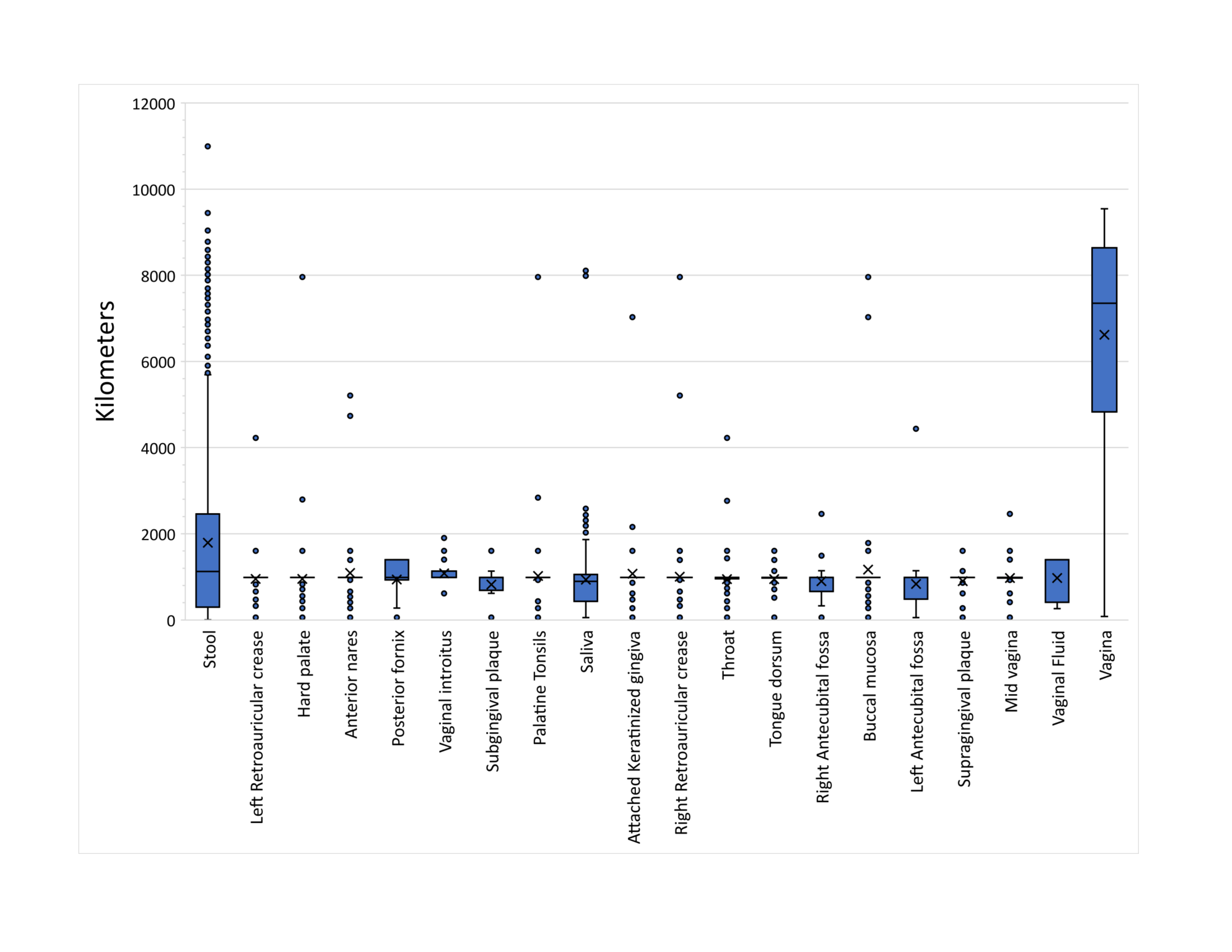

Supplement: Supplementary file 2 [file Image_2.tiff]

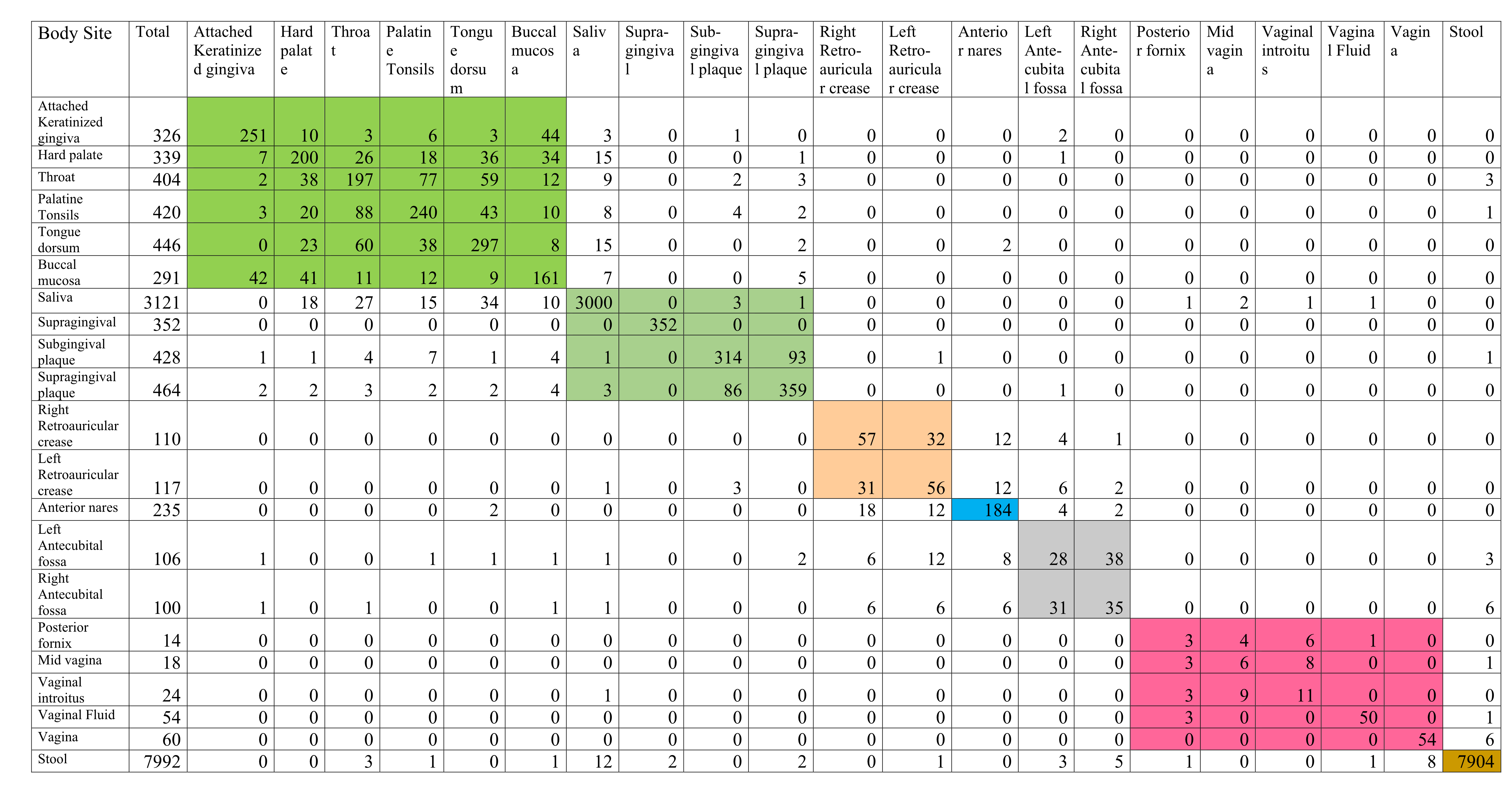

Supplement: Supplementary file 3 [file Image_3.tiff]
